# Maxillary Sinus Lift Procedures: An Overview of Current Techniques, Presurgical Evaluation, and Complications

**DOI:** 10.7759/cureus.49553

**Published:** 2023-11-28

**Authors:** Abdulrahman M Alshamrani, Mazen Mubarki, Abdulelah S Alsager, Hussam K Alsharif, Saud A AlHumaidan, Ahmad Al-Omar

**Affiliations:** 1 Dentistry, College of Dentistry, King Saud University, Riyadh, SAU; 2 Surgery, King Saud University, Riyadh, SAU

**Keywords:** sinus lifting procedure, complications, schneiderian membrane, anatomy, bone augmentation, perforation, maxillary sinus

## Abstract

A maxillary sinus lift procedure is indicated if a dental implant needs to be placed in the posterior maxilla with limited bone available to accommodate a dental implant. Both open and closed sinus lifting procedures are reliable approaches for increasing the bone volume needed to support proper implant positioning. However, these methods can lead to several complications. In addition to the general complications commonly linked to oral surgery, such as swelling or hematoma, the primary complication in open sinus lifting is typically the perforation of the Schneiderian membrane during osteotomy. Detailed and extensive presurgical evaluation is crucial to minimize such complications. The objective of this study was to delineate contemporary trends in sinus lift surgery, with a specific emphasis on different techniques of sinus lift procedure, anatomical and surgical factors, presurgical evaluation, bone grafting, and the practical implications of these factors in implant dentistry cases involving a deficient posterior maxilla. In conclusion, while both osteotome and lateral window techniques can assist clinicians in addressing the complexities of implant placement in a deficient posterior maxilla, bone height before implantation remains a critical factor in determining the success and longevity of implants.

## Introduction and background

The maxillary sinus is the largest among all the paranasal sinuses [1]. Two types of bone resorption occur when patients lose their maxillary posterior teeth. The first type is centripetal resorption, which is a natural result of the bone remodeling process following tooth loss. The second type is resorption, caused by the pneumatization of the sinus cavity towards the alveolar crest [2]. Both types of resorption often lead to a reduced amount of bone available for placement of dental implants, necessitating a regenerative procedure known as a maxillary sinus lifting procedure. Sinus lifts are regarded as a safe treatment option with a lower risk of complications [3,4]. The primary goal of this intervention is to create enough bone height and width to facilitate the proper placement of dental implants. This goal can be achieved using either a one-stage or two-stage technique. The one-stage technique inserts dental implants simultaneously with the sinus augmentation procedure. With the two-stage technique, bone augmentation is performed during the initial surgical procedure, and the dental implants are placed later once the necessary bone volume has been established [5]. The traditional sinus lift procedure, initially explained by Tatum H [3,6] in the 1970s, involved a combination of incisions. This combination included a crystal incision along with mesial and distal vertical incisions, allowing for the elevation of a buccal flap to expose the outer bone wall of the sinus. Subsequently, a trapdoor osteotomy (window) was made in the lateral bone wall, providing access to the Schneiderian membrane and the sinus cavity. Schneiderian membrane is a membrane that forms the lining of the inner aspect of the maxillary sinus. The membrane was then meticulously dissected and lifted in an apical direction, with particular care taken to preserve its integrity. This displacement of the membrane created space for the graft material. Bone replacement grafts in maxillary sinus lift procedures encompass a variety of materials. These include autologous bone, which can be sourced from the mandibular ramus, chin, iliac crest, or other intraoral locations, as well as bone substitutes, synthetic biomaterials, or combinations of these substances [7,8]. In cases where patients have sufficient remaining bone height, it is possible to augment the sinus floor using a less-invasive method known as the trans-alveolar approach, which involves the use of the osteotome technique. This technique, first employed by Summers RB [7] in 1994, allows sinus floor augmentation without the need for extensive surgery. However, complications are possible during maxillary sinus lift surgery. The most frequently encountered intraoperative complication in maxillary sinus lift procedures is the perforation of the sinus membrane. Other potential complications include postoperative infection, sinusitis, graft exposure, graft loss, edema (swelling), seroma formation (accumulation of fluid), bleeding, and membrane exposure [9-12]. The objective of this study is to review the maxillary sinus lift procedure, encompassing preoperative assessment, surgical techniques, bone grafting materials, and possible complications.

## Review

Anatomy

The maxillary sinus holds approximately 12-15 mL of air in adults [13]. It has a pyramidal shape, with its base near the nasal cavity, the upper part serving as the orbital floor, and the tip toward the zygomatic bone [14]. An oval or slit-shaped drainage opening, known as the ostium, functions as an overflow opening and is positioned in the upper part of the inner wall [14,15]. The space between the semilunar hiatus and the nasal floor can range from 18 to 35 mm, with an average of 25.6 mm [16]. The position of the ostium minimizes the chances that it will be blocked during augmentation procedures [17]. The base of the maxillary sinus extends from the premolar or canine region anteriorly and to the maxillary tuberosity posteriorly, often reaching its lowest point near the first molar area [18]. In dentate adults, the maxillary sinus floor is the thickest of its walls and lies approximately at the same level as the nasal floor. However, in patients who have lost their teeth (edentulous), it is typically situated about 1 cm below the nasal floor. Septa within the sinus is composed of cortical bone and can be found both horizontally and vertically within the sinus floor [19,20]. Some studies have observed septa in approximately 25%-31.7% of maxillary sinuses [21,22], and these septa can range from 2.5 to 12.7 mm in length and be in various locations within the maxillary sinus [11]. Notably, there tend to be more septa in edentulous or atrophic (reduced in size) ridges than in partially edentulous or nonatrophic arches [19,21].

Blood supply and innervation

The primary branches of the maxillary artery, which supply blood to the bony walls and membrane of the sinus, include the posterior superior alveolar artery, inferior orbital artery, greater palatine artery, and sphenopalatine artery. It is essential to note that the locations of the inferior orbital artery and the posterior superior alveolar artery are crucial considerations in surgical planning, as any damage to these arteries can result in bleeding complications [23,24]. These two arteries eventually join together, forming a dual arterial arcade that encircles the maxillary sinus [25]. This connection can occur in either an extraosseous manner, typically located about 23-26 mm away from the alveolar ridge, or an endosseous fashion, positioned approximately 16.4-19.6 mm from the alveolar margin [24]. It is noteworthy that the dental branch of the posterior superior alveolar artery consistently exhibits an endosseous connection with the inferior orbital artery in all dissected anatomical cases; however, this connection is visible on radiographs in only 50% of cases [25-27]. The innervation of the maxillary sinus is outlined in Table 1. It represents a distinct connection between the venous system of the maxillary sinus and the cavernous sinus, which is significant because it can potentially serve as a pathway for infections spreading from the sinus to the brain [28-30]. 

**Table 1:** The nerve supply to the maxillary sinus. PSA: Posterior superior alveolar; MSA: Middle superior alveolar; ASA: Anterior superior alveolar; IO: Infra orbital; GP: Greater palatine. Adapted from [28-30]

Nerve	Area of supply
PSA + MSA nerves	Posterior sinus wall
ASA nerve	Anterior sinus wall
IO nerve	The superior wall of the sinus and part of the medial wall
GP nerve	Inferior wall of the sinus and ostium

Presurgical evaluation

The presurgical evaluation is preliminarily done through CT or cone-beam computed tomography (CBCT) scans. This evaluation determines essential parameters such as membrane thickness, presence of sinus septa, residual bone height, and presence of teeth. The elevation of the maxillary sinus floor carries a risk of jeopardizing the sinus physiology, and a careful and thorough CBCT evaluation before the procedure can reduce the chances of intra-operative and post-operative complications [31,32]. The maxillary sinus is considered healthy when the mucous composition is normal, mucociliary clearance is efficient, and the sinus ostium is patent. These criteria are significant because a healthy maxillary sinus is less likely to develop postsurgical complications, even in the event of a small procedural error, such as a minimal perforation [33].

Risk of perforation

The risk of perforation can be associated with irregularity in the membrane thickness, sinus septa, the angle between the buccal and palatal wall, and existing tooth implants or tooth roots adjacent to the sinus [34].

The Schneiderian membrane

The Schneiderian membrane is an important parameter during the presurgical analysis [35,36]. Membrane thickness of up to 2 mm is considered physiological and favorable; however, thickness exceeding 5 mm is associated with sinus ostium obstruction. Recent CBCT studies indicate that 1 mm is a physiological value and 4 mm is pathological [37-40].

Sinus septa

In approximately 38% of all cases, sinus septa (or Underwood's septa) are found inside the maxillary sinus. Depending on their shape, position, and development, they may threaten membrane integrity during sinus floor elevation, and the presence of these anatomical variations can enhance the risk of perforation [17,38,41]. The development of Underwood's septa should be considered in judging the complexity of sinus floor elevation during surgery. If the sinus septum runs transversely, surgery is straightforward, but if it is longitudinal or incomplete, the procedure may become more difficult during membrane elevation [42].

Alveolar-antral artery

**Figure 1:**
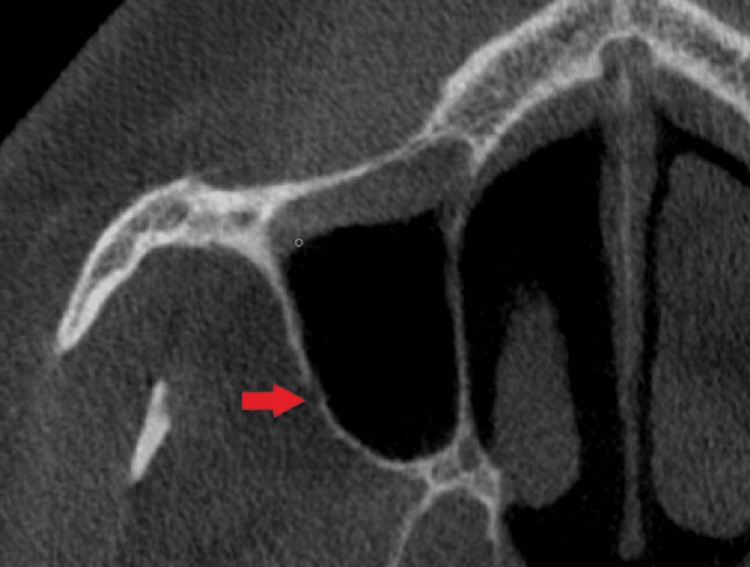
CBCT showing both the diameter and course of the alveolar-antral artery. CBCT: Cone-beam computed tomography. Source: [43]

An intraosseous anastomosis, the alveolar-antral artery, is always present between the posterior superior alveolar artery and the infraorbital artery. However, an extraosseous anastomosis exists in only 44% of cases. Hemorrhage of the alveolar-antral artery is a common complication in sinus lifting procedures. To avoid this, a posterior approach to the bone antrostomy has been suggested. Planning should include a careful evaluation of CBCT to ascertain the course of the artery. Another important consideration is the artery's diameter. If the diameter is less than 1 mm, or if the artery cannot be detected radiographically, the likelihood of severe complications during surgery is minimal. Conversely, if the diameter is 2-3 mm or greater, the risks of hemorrhage and the need to ligate the artery increase [34]. Both the diameter and course of the artery are evaluated through CBCT, as shown in Figure 1 [43].

Presence of teeth

**Figure 2:**
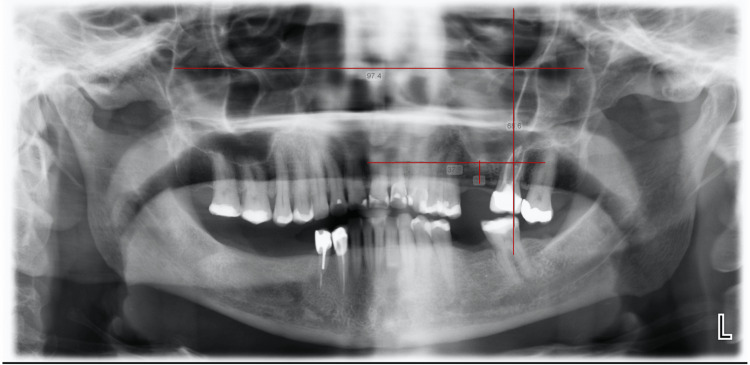
A panoramic image showing reference lines drawn and perpendicular distances measured from the crest of the bone to the maxillary sinus floor. Source: [46].

The resorption of the alveolar ridge and the maxillary sinus pneumatization are both profoundly influenced by the loss of posterior teeth [44]. When a close relation between the sinus membrane and tooth roots has been detected, especially in the case of a single posterior missing tooth, the perforation risk increases [45]. However, the probability of perforation decreases when two adjacent teeth are missing. This decreased probability could be due to the presence of sinus pneumatization in a small area with an irregular sinus floor shape. Figure 2 shows the relationship between the extraction of teeth and pneumatization of the maxillary sinus [46].

Residual alveolar ridge height

Residual alveolar ridge height has been suggested to significantly influence membrane thickness [47] and the success of implant therapy over time [9]. Alveolar ridge height also plays a major role in implant survival rates [48]. According to some studies [49,50], a pre-implant bone height of less than 5 mm is associated with a decreased survival rate. These findings indicate that a higher success rate could be achieved with greater alveolar bone height.

General considerations

Generally, sinus lifting is indicated with a residual bone height of 10 mm or less (including leaving a space of 1 to 2 mm of bone between the implant apex and the sinus floor level) [48]. The two basic methods for the sinus lifting procedure are the trans-alveolar (crestal osteotome) and the lateral window [51]. If more than 5 mm of bone height is present, the crestal osteotome is the treatment of choice [52]. However, if the ridge height is severely reduced, the use of a lateral window is indicated. This technique can aid in achieving a height of up to 9 mm, which is enough to compensate for the bone shortage [48]. Factors affecting the prognosis of the maxillary sinus lifting procedure are demonstrated in Table 2.

**Table 2:** A summary of factors affecting the prognosis of maxillary sinus lifting procedures. Source: [34]

Prognosis	The Schneiderian membrane	Sinus septa	Alveolar-antral artery	Presence of teeth (from second premolar to second molar)	Residual alveolar ridge height
Most favorable	1-2 mm	Absence of septa	Diameter less than 1 mm or not detectable radiographically	Totally missing teeth	Higher than 4 mm
Normal	0.8-1.49 mm, 2.01-2.99 mm.	Presence of one complete and transverse sinus septum	1-2 mm	Two adjacent missing teeth	2-4 mm
Least favorable	Less than 0.80 or more than 3 mm	Presence of one or more incomplete or longitudinal sinus septa	More than 2 mm	Single missing tooth	Less than 2 mm

Surgical techniques

The maxillary sinus lift procedure has gained widespread acceptance to reduce postoperative complications in cases of limited bone height in the posterior maxillary alveolar ridge [48]. This procedure is typically advised in cases with bone height in the posterior maxilla of 10 mm or less [53]. Tatum H [54] introduced the initial lateral window procedure in 1975. This technique involves surgically creating an opening in the lateral wall of the sinus and then gently lifting the Schneiderian membrane to facilitate placing the implant(s) of suitable length. The use of the lateral approach is particularly valuable in cases of substantial bone deficits because it allows an increase in vertical bone height by more than 9 mm [48]. The osteotomy can be executed by utilizing either a high-speed handpiece or precise piezoelectric instruments. Using a piezoelectric tip to prepare the window greatly reduces the likelihood of membrane perforation and results in an overall safer procedure [54-56]. In 1994, Summers RB [7] was the first to employ the osteotome approach. This technique involves a transalveolar elevation of the maxillary sinus floor. This approach offers several advantages, including efficient surgical procedures, reduced surgical duration, fewer complications, lower postoperative discomfort, and increased patient satisfaction. Furthermore, the osteotome approach typically increases vertical bone height from 3 to 9 mm [52,57,58]. To optimize the results of lifting the maxillary sinus, various minimally invasive strategies have been developed to provide an increased level of patient satisfaction [48]. The antral membrane balloon elevation is a minimally invasive approach designed to gradually lift the Schneiderian membrane while ensuring its preservation. The membrane is carefully separated by applying gentle and sustained pressure while inflating a latex balloon. This method is considered relatively safer, with minimal postoperative bleeding, pain, or discomfort [59,60]. Large-scale longitudinal studies are required to establish the procedure's clinical effectiveness and long-term outcomes [61]. Recently, a new bioactive kinetic screw bone implant model efficiently accomplishes both autogenous grafting and sinus augmentation while also securing the implant in a single procedure. When the vertical bone height in the planned implant site is less than 4 mm, an additional surgical step is undertaken to harvest bone and enhance its availability [62-66]. Experimental studies conducted on synthetic maxillary bone and sinus have successfully demonstrated the feasibility and simplicity of this innovative technique. However, more studies are needed to further evaluate this technique [67]. The selection of the appropriate surgical technique for sinus lift procedures primarily depends on the height of the existing pre-implant bone. The transcrestal approach tends to be preferred when the residual bone height is greater than 5 mm. In cases where the residual bone height is 5 mm or less, the lateral window approach is considered more suitable [7,58,68-70].

Bone grafting materials

Bone grafts can be used to promote bone formation and maxillary sinus augmentation can be accomplished through the use of autografts, allografts, xenografts, alloplastic material, and growth factors [71]. Autogenous grafts obtained from the same individual are considered the gold standard because of their osteogenic capacity and osteoconductive and osteoinductive properties. Autogenous grafts also heal quickly and have strong resistance to infections. However, the increased morbidity and unpredictable reabsorption associated with autografts have led to the development and use of synthetic substitutes. Allogenic grafts, obtained from another individual in the same species, have only osteoconductive and osteoinductive capabilities. Xenografts, obtained from different species, possess only osteoconductive capability. Alloplastic grafts, whether natural or synthetic, are solely osteoconductive biomaterials [51,72]. Adding xenografts to autogenous grafts improves volumetric stability in the sinus augmentation procedure [73]. All graft types can be prepared in different forms, such as large blocks or streaky gels. Some authors have suggested performing sinus lift procedures without grafting materials by utilizing coagulated blood as a scaffold to form new bone. However, this technique was not evaluated alongside appropriate control procedures, and the results were not reproducible [4]. A different approach was introduced in managing cases using platelet-rich plasma or plasma rich in growth factors, with or without grafting biomaterials. The preparation of platelet-rich plasma involves the use of citrate in blood samples to prevent coagulation and maintain a liquid form. To prepare the gel form, thrombin and/or calcium chloride are added to induce fibrin polymerization [74]. Platelet-rich fibrin is considered a second-generation platelet concentrate, offering additional advantages such as enhanced healing capabilities, low cost, and ease of handling. Platelet-rich fibrin can improve new bone formation, and significant results can be obtained after a sinus lift procedure [75]. The use of platelet-rich fibrin can also improve implant stability and the osseointegration process [76]. Platelet-rich fibrin is currently a trend in the management of sinus lift procedures and is considered superior to first-generation concentrates [77].

Complications of maxillary sinus lift procedure

Just as with any other surgical procedure, a sinus lift is associated with various complications, including intraoperative complications, acute postoperative complications, and chronic postoperative complications [78].

Intraoperative Complications

Common complications that can occur during maxillary sinus graft surgery are the perforation of the Schneiderian membrane, penetration into the sinus or nasal cavity, bleeding, damage to the adjacent teeth, bone fracture, perforation of the alveolar bone, inadequate initial implant stability, incorrect placement or alignment of the implant, blockage of the opening to the maxillary sinus, and accidental swallowing of surgical instruments [78].

Tearing of the Schneiderian membrane: Tearing of the Schneiderian membrane is the most frequently encountered complication during maxillary sinus graft procedures. The incidence of this complication falls within the range of 20%-44% when the lateral window approach is used [51]. Ardekian L et al. [79] reported that perforation of the sinus membrane happened in 85% of cases with a residual ridge measuring 3 mm, whereas in cases with a 6-mm residual ridge, membrane perforation only occurred in 25% of cases. Minor perforations may not necessitate treatment, but in the event of a significant perforation, the procedure should either be halted, or a collagen membrane should be applied to repair the perforation. If the procedure is stopped, a subsequent attempt should not be made for another 4-6 months [10].

Bleeding: The maxillary sinus region contains a network of blood vessels, with the primary vessel being the maxillary artery. This artery gives rise to multiple branches, including the intraorbital artery, the anterior superior palatine artery, and the posterior superior alveolar artery, that supply blood to the sinus cavity and the adjacent tissues and structures. Numerous connections (anastomoses) are typically observed between the posterior superior alveolar artery and the infraorbital artery within the lateral bony wall of the sinus. These connections play a crucial role in ensuring adequate blood circulation in this region [80]. Bleeding can occur if arteries are damaged during the preparation of the lateral window. To mitigate this risk, it is advisable to identify the location of the artery prior to surgery using CBCT [2]. To address a severed vessel, various methods have been suggested, including applying strong pressure, directly tying off the vessel, introducing particulate bone graft into the arterial canal, using bone wax, smoothing the area with burs, and employing electrocautery. Additionally, having the patient sit upright can help reduce blood flow by 38%, aiding in the control of bleeding [28].

Acute Postoperative Complications

Immediate postoperative complications include discomfort, inflammation, swelling, infection affecting both the surgical area and the sinus, sinusitis, bone loss, bleeding, bruising around the mouth and nose, and hematoma (particularly hemosinus). Other potential issues include the presence of emphysema, wound opening, graft loss, fixture displacement or loss, the formation of an oroantral fistula, benign paroxysmal positional vertigo, and transient or permanent numbness in the palate [78].

Chronic Postoperative Complications

While implant periapical lesions are infrequent in the maxilla, they can arise in clinical situations in which excessive heat is generated during the drilling process. When the bone is assessed as hard, a longer time gap (at least one minute) between drilling stages is advised. Additionally, utilizing chilled saline instead of the standard room-temperature saline solution can be beneficial [78].

## Conclusions

In conclusion, maxillary sinus augmentation is an effective preprosthetic method for enhancing the edentulous posterior maxilla. A thorough presurgical evaluation of sinus anatomy significantly lowers the likelihood of complications. Using growth factors and stem cells is a promising technique to improve graft maturation time, although additional clinical research is required to fully assess their advantages.
